# Advances in the Study of Schistosomiasis: The Postgenomic Era

**DOI:** 10.1155/2013/849103

**Published:** 2013-03-05

**Authors:** Cristina Toscano Fonseca, Sergio Costa Oliveira, Andrea Teixeira-Carvalho, Rashika El Ridi

**Affiliations:** ^1^Centro de Pesquisas René Rachou, Fundação Oswaldo Cruz, 30190-002 Belo Horizonte, MG, Brazil; ^2^Department of Biochemistry and Immunology, Federal University of Minas Gerais, 31270-901 Belo Horizonte, MG, Brazil; ^3^Zoology Department, Faculty of Science, Cairo University, Giza 12613, Egypt

Schistosomiasis remains a serious public health problem in 76 countries and territories of the world, with more than 200 million individuals infected, causing annual losses of 10.4 million in DALYs (disability adjusted to life years). Although effective drugs against schistosomiasis are used in disease control programs, the prevalence of the disease remains unaltered, and new diseases foci and acute outbreaks are observed worldwide. Also, drug resistance by the parasites is a concern, and new drugs and alternative control measures need to be developed.

An effective vaccine against schistosomiasis is an alternative and desirable tool to help in the disease control; however, the complex schistosome life cycle and the complexity of its interaction with the host immune system turn vaccine development into a difficult task. Neither drug development nor vaccine development is sufficient to ensure the success of schistosomiasis control programs. As a matter of fact, the combination of an effective vaccine together with chemotherapy is the strategy of choice. Many studies also demonstrate that without a more sensitive diagnosis test, able to detect individuals with low parasite burden, no control strategy will achieve the desirable result which nowadays is diseases elimination.

The access to the genome from *Schistosoma mansoni*, *Schistosoma japonicum,* and *Schistosoma haematobium* recently published is expected to increase rapidly our understanding of the parasite biology and also accelerate the development of new drugs, vaccine, and diagnosis methods by helping in the identification of new targets.

In this special issue, a review by C. T. Fonseca et al. gives an update on how parasite genomes together with bioinformatics tools have helped in vaccine and diagnosis development in the last few years. Since the parasite surface represents an interesting target for the host immune system, this review was focused on parasite tegument as a source of antigens.

Although the genome from the three most socioeconomically important schistosome species had already been published, many questions continue to challenge researchers. The review by L. A. Nahum and coworkers discusses the advances on schistosome genomics and transcriptomics studies and point out the gaps and future directions in this research field.

An example on how parasite genome database helps in understanding the parasite biology is presented in the paper by R. V. Pereira and colleagues. In their article, using *in silico* analysis, they identified in the *S. mansoni* genome orthologs of the mammalian Sentrin/SUMO-specific proteases (SENPs) 1 and 7, which are involved in many cellular processes. The *S. mansoni* SENPs transcriptional profile as well as its structural conservation was analyzed.

Just as important as understanding the biology of the parasite is deciphering how the host immune system deals with schistosome infection and its relation to the pathogenesis of the disease. Clinically schistosomiasis is characterized by two distinct phases: the acute and the chronic phase. The acute phase is commonly observed in individuals living in nonendemic area, while chronic phase is observed in endemic-area residents. 

D. Silveira-Lemos et al. evaluated in their article immunological parameters in patients with acute schistosomiasis before and after praziquantel treatment. Interesting differences are demonstrated by the authors between noninfected, acute, and treated group, and a role for CD8+ T cells as a source of cytokines in acute patients is pointed by this study.

The cytokine and chemokine profile observed in PBMCs from individuals infected with *S. mansoni* in response to specific stimulation and its correlation with different degrees of periportal fibrosis is described by R. P. de Souza and colleagues in their research article. Their results indicate potential biomarkers for the progression of liver pathology due to schistosomiasis.

Many schistosome antigens have been described to modulate inflammatory diseases such as asthma. A. M. B. Bafica et al. evaluated in their article the impact of the *S. mansoni* antigens: Sm29, TSP-2, and PIII in PBMCs culture from cutaneous leishmaniasis patients stimulated with *L. braziliensis* antigen. Their results demonstrated that schistosome antigens induce a modulatory phenotype with decreased monocyte activation and increased expression of T-lymphocyte modulatory molecules.

*Trichobilharzia *spp. infection results in bird schistosomiases, which causes severe pathogenic impact in birds and frequently results in cercarial dermatitis in humans. In their article, L. Lucie and H. Petr review the pathogenesis of bird schistosomiasis and the immune response triggered by *Trichobilharzia *spp. infection with emphasis on the new species *T. regent*.

We hope you enjoy reading this special issue!

*Cristina Toscano Fonseca* *Cristina Toscano Fonseca* *Sergio Costa Oliveira* *Sergio Costa Oliveira* *Andrea Teixeira-Carvalho* *Andrea Teixeira-Carvalho* *Rashika El Ridi* *Rashika El Ridi*

